# A novel small molecule ameliorates ocular neovascularisation and synergises with anti-VEGF therapy

**DOI:** 10.1038/srep25509

**Published:** 2016-05-05

**Authors:** Rania S. Sulaiman, Stephanie Merrigan, Judith Quigley, Xiaoping Qi, Bit Lee, Michael E. Boulton, Breandán Kennedy, Seung-Yong Seo, Timothy W. Corson

**Affiliations:** 1 Eugene and Marilyn Glick Eye Institute, Department of Ophthalmology, Indiana University School of Medicine, Indianapolis, Indiana 46202, United States of America; 2 Department of Pharmacology and Toxicology, Indiana University School of Medicine, Indianapolis, Indiana 46202, United States of America; 3 Department of Biochemistry, Faculty of Pharmacy, Cairo University, Cairo, Egypt; 4 School of Biomolecular and Biomedical Science, Conway Institute, University College Dublin, Dublin 4, Ireland; 5 College of Pharmacy, Gachon University, Incheon 406-840, South Korea; 6 Department of Biochemistry and Molecular Biology, Indiana University School of Medicine, Indianapolis, Indiana 46202, United States of America

## Abstract

Ocular neovascularisation underlies blinding eye diseases such as retinopathy of prematurity, proliferative diabetic retinopathy, and wet age-related macular degeneration. These diseases cause irreversible vision loss, and provide a significant health and economic burden. Biologics targeting vascular endothelial growth factor (VEGF) are the major approach for treatment. However, up to 30% of patients are non-responsive to these drugs and they are associated with ocular and systemic side effects. Therefore, there is a need for small molecule ocular angiogenesis inhibitors to complement existing therapies. We examined the safety and therapeutic potential of SH-11037, a synthetic derivative of the antiangiogenic homoisoflavonoid cremastranone, in models of ocular neovascularisation. SH-11037 dose-dependently suppressed angiogenesis in the choroidal sprouting assay *ex vivo* and inhibited ocular developmental angiogenesis in zebrafish larvae. Additionally, intravitreal SH-11037 (1 μM) significantly reduced choroidal neovascularisation (CNV) lesion volume in the laser-induced CNV mouse model, comparable to an anti-VEGF antibody. Moreover, SH-11037 synergised with anti-VEGF treatments *in vitro* and *in vivo*. Up to 100 μM SH-11037 was not associated with signs of ocular toxicity and did not interfere with retinal function or pre-existing retinal vasculature. SH-11037 is thus a safe and effective treatment for murine ocular neovascularisation, worthy of further mechanistic and pharmacokinetic evaluation.

Neovascular eye diseases are a major cause of blindness throughout life. Between 6% and 18% of childhood blindness is attributable to retinopathy of prematurity (ROP)^1^. The disease is estimated to cause vision loss in 1300 children a year in the USA, and severe visual impairment in a further 500^2^. Meanwhile, proliferative diabetic retinopathy (PDR) affects an estimated 17 million adults of working age worldwide, or 7% of the growing diabetic population^3^. Finally, almost 2 million elderly Americans are affected by “wet” age-related macular degeneration (AMD)^4^. Wet AMD has an estimated loss of productivity burden of $5.4 billion annually in the United States^5^. These diseases are characterised by the development of new blood vessels in the eye: either retinal (in ROP and PDR) or choroidal neovascularisation (CNV), where blood vessels grow into the sensory retina, causing hemorrhage and severe vision impairment^6^.

A standard treatment for these blinding diseases involves biologic drugs (bevacizumab, ranibizumab and aflibercept) that target vascular endothelial growth factor (VEGF), the most dominant angiogenic mediator in the eye^7^,^8^,^9^. These biologics successfully slow or even reverse vision loss in about 70% of wet AMD patients^10^, and are now being used for ROP and PDR. However, repeated intravitreal injections of anti-VEGF drugs can be associated with ocular and systemic side effects such as ocular haemorrhage and stroke^9^,^11^,^12^. Moreover, about 30% of wet AMD patients are non responsive to anti-VEGF treatments^13^. Therefore, there is an unmet need to develop new antiangiogenic molecules to complement and combine with these existing approaches. Such novel therapies might also be useful for other neovascular diseases, including cancers.

Natural products continue to provide a rich source for the discovery of small molecule drugs^14^,^15^. A select subset of compounds has been tested specifically in the context of ocular angiogenesis^16^. In particular, the structural, chemical and pharmacological diversity of homoisoflavonoids has attracted researchers interested in developing small molecule therapies for various diseases^17^. We^18^ and others^19^,^20^,^21^ have previously tested the natural homoisoflavonoid, cremastranone, for its antiangiogenic potential *in vitro* and *in vivo* in ocular disease models. We recently developed a synthetic derivative of cremastranone, named SH-11037 (Fig. 1a), using a cell-based structure-activity relationship analysis^18^. SH-11037 was more potent than the parent compound, cremastranone, with about 10-fold antiproliferative selectivity towards human retinal endothelial cells (HRECs) over macrovascular endothelial cells, and had negligible effects on other ocular cell types. Moreover, SH-11037 inhibited HREC proliferation, migration, and tube formation in a concentration-dependent manner, without inducing apoptosis. Together, these data provide a strong *in vitro* indication of SH-11037’s antiangiogenic activity without cytotoxicity^18^.

A detailed evaluation of the antiangiogenic therapeutic potential and safety of SH-11037 for ocular neovascularisation has not been performed previously. Here, we explored SH-11037’s pharmacological activity using a choroidal angiogenesis model *ex vivo*, a simple ocular neovascularisation model in zebrafish larvae, and *in vivo* in the laser-induced choroidal neovascularisation (L-CNV) mouse model as a single treatment and in combination with the standard-of-care anti-VEGF antibody. We also assessed intraocular toxicity of this compound in mice. We show that SH-11037 has a strong antiangiogenic potential on CNV in the absence of ocular toxic effects, which could make it an alternative or additive therapy to existing anti-VEGF drugs for treatment of neovascular diseases in the eye and other tissues.

## Results

### SH-11037 inhibits choroidal neovascularisation *ex vivo* in the choroidal sprouting assay

**Figure 1:**
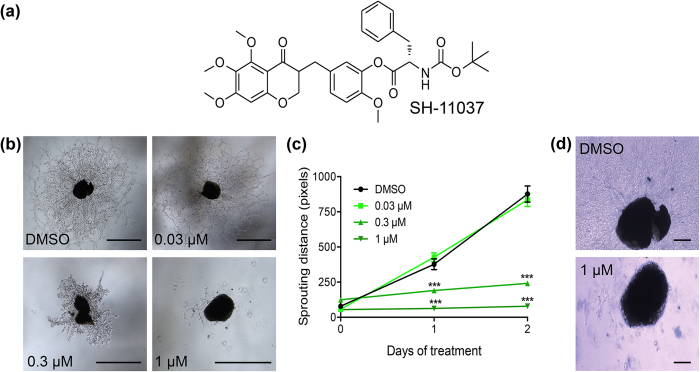
SH-11037 inhibits choroidal sprouting in a concentration-dependent manner without affecting cell viability. (**a**) Structure of SH-11037. (**b**) Representative images of choroidal sprouts formed 48 hours after treatment with indicated SH-11037 concentrations or DMSO control, scale bars = 1000 μm. (**c**) Quantification of sprouting distance from the edge of the choroid tissue piece to the end of the sprouts averaged from four perpendicular directions using ImageJ software. ***P < 0.001, repeated measures two-way ANOVA, Dunnett’s post hoc test, Mean ± s.e.m. n = 4 from two independent experiments. (**d**) Representative images of trypan blue stained choroidal sprouts 48 hours after treatment with DMSO and 1 μM SH-11037 to assess cell viability, scale bars  =  1000 μm.

To investigate the effect of SH-11037 on choroidal angiogenesis, we first tested different concentrations of SH-11037 on the sprouting of mouse choroidal tissues *ex vivo*. After 48 hours of incubation with SH-11037, the ability of the choroidal tissues to form sprouts was significantly inhibited in a concentration-dependent manner compared to the vehicle treated controls (Fig. 1b,c). Sprouting was absent at the highest concentrations tested. Trypan blue staining for viable cells indicated the absence of significant cell death in SH-11037 treated wells compared to DMSO controls (Fig. 1d); this is consistent with the lack of apoptosis induced by SH-11037 in HRECs^18^.

### Systemic administration of SH-11037 inhibited ocular angiogenesis in zebrafish larvae

**Figure 2:**
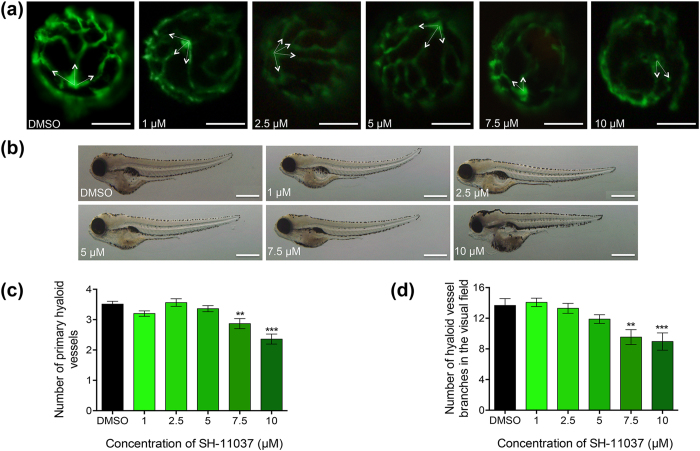
SH-11037 significantly inhibits ocular developmental angiogenesis in zebrafish larvae. (**a**) Representative epi-fluorescence images of hyaloid vessel (HV) morphology on lenses dissected from drug-treated 5 days post fertilisation (dpf) *Tg*(*fli1:EGFP*) larvae. A significant reduction in primary HV branch number is observed at 10 μM SH-11037 (white arrows), scale bars = 50 μm. (**b**) Representative bright-field images of 5 dpf larval morphology following SH-11037 treatment. Some larvae treated with 10 μM SH-11037 demonstrated pericardial and yolk sac oedema. Scale bars = 500 μm. (**c**) Quantification of the dose-dependent inhibition of primary HV development in 5 dpf *Tg*(*fli1:EGFP*) larvae treated from 2 dpf with 1–10 μM SH-11037. (**d**) Quantification of the dose-dependent inhibition of SH-11037 on number of hyaloid vessel branches in treated larvae compared to DMSO control. Up to a 40% reduction in primary HV and vessel branches is observed in response to 10 μM SH-11037. ***P <0.01, ***P < 0.001, one-way ANOVA with Dunnett’s post hoc tests. Mean ± s.e.m., n ≥ 3.

To evaluate the antiangiogenic activity of SH-11037 in an intact vertebrate model system, we analysed the inhibition of primary hyaloid vessel (HV) and hyaloid vessel branch development in the eyes of *Tg*(*fli1:EGFP*) zebrafish larvae^22^. The number of vessels and branches attached to the larval lens at 5 days post fertilisation (dpf) was quantified. SH-11037 treatment resulted in a dose-dependent inhibition of HV development with 10 μM SH-11037 significantly attenuating the number of primary HV and branches, consistent with an antiangiogenic activity in the eye. Qualitatively, SH-11037 showed about 20 and 40% reduction in the overall HV branch pattern at 7.5 and 10 μM, respectively (Fig. 2a,c,d). Overall zebrafish morphology at tested doses of SH-11037 appeared normal with some pericardial and yolk sac oedema evident at 10 μM (Fig. 2b). Lethality was observed in less than 5% of the larvae at 10 μM and such larvae were omitted from analysis. Additionally, no difference in numbers of intersegmental (non-ocular) vessels was observed (mean ± s.e.m., n = 23−30): 28.6 ± 0.2 at 5 μM, 28.5 ± 0.2 at 7.5 μM, 28.3 ± 0.2 at 10 μM and 28.3 ± 0.2 for the DMSO control, suggesting the absence of effects on pre-existing non-ocular vessels.

### Intravitreal injection of SH-11037 does not cause acute or chronic ocular toxic effects

**Figure 3:**
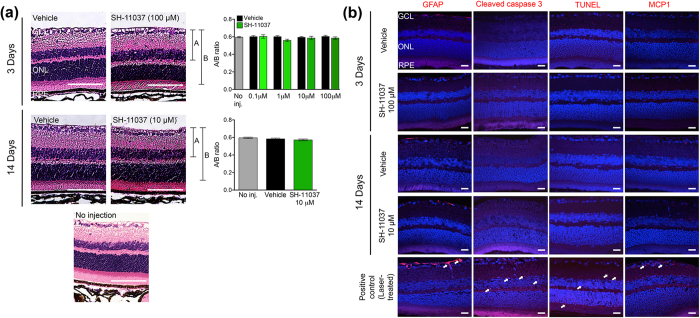
Intravitreal injection of SH-11037 does not cause ocular toxicity after 3 and 14 days. (**a**) Representative images of H&E stained retinas from vehicle and SH-11037 treatments, and uninjected control, scale bars = 100 μm, and quantification of the indicated A/B ratio show the absence of short- or longer-term changes in retinal layers at any given concentration of SH-11037. *P* > 0.05, Student’s t-tests, Mean ± s.e.m., n = 8 eyes/treatment. (**b**) Immunofluorescence staining revealed no differences between SH-11037 treatment and vehicle control in GFAP, cleaved caspase 3, TUNEL, and MCP1 (each red) as markers of retinal injury, apoptosis, or inflammation, respectively. Laser injured eyes were stained for the same markers as a positive control; white arrows indicate the increased expression of respective markers. Nuclei are stained with DAPI (blue). Scale bars = 20 μm. GCL, ganglion cell layer; ONL, outer nuclear layer; RPE, retinal pigment epithelium.

Before evaluating the therapeutic potential of SH-11037 injections in mammalian eyes, we first investigated possible toxicity. We examined signs of acute (3 days post injection) and chronic (14 days post injection) retinal toxicity of intravitreally injected SH-11037 up to 100 μM final concentration in the eye. Haematoxylin and eosin (H&E) stained retinal sections of SH-11037 treated eyes revealed no histological changes compared to vehicle treatment and no injection control eyes (Fig. 3a). Quantification of retinal thickness demonstrated the absence of morphological changes between SH-11037 treatments compared to vehicle-treated and uninjected eyes at 3 and 14 days post injections. To further investigate any toxic effects of SH-11037 beyond morphology, retinal sections were stained for glial fibrillary acidic protein (GFAP), cell death markers (cleaved caspase 3, TUNEL) and monocyte chemotactic protein-1 (MCP1) to examine any signs of retinal injury, apoptosis or inflammation, respectively. Neither short- nor longer-term SH-11037 treated retina showed any significant increases of GFAP, cleaved caspase 3, TUNEL staining and MCP1 compared to vehicle-treated controls (Fig. 3b). These data suggest the absence of toxic effects of SH-11037 on retina examined 3 and 14 days post injections.

### SH-11037 does not affect retinal function and pre-existing vessels

**Figure 4:**
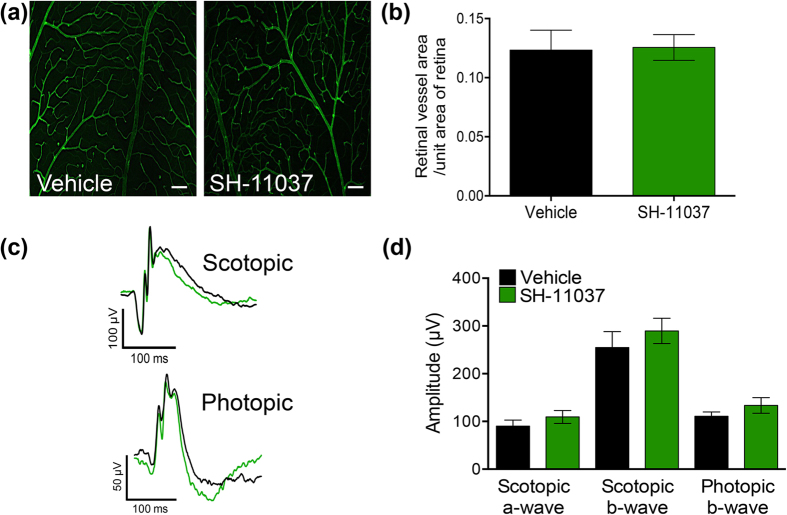
SH-11037 does not interfere with retinal function and pre-existing vasculature. (**a**) Isolectin-stained retinal vasculature does not differ between 100 μM SH-11037 and vehicle treated control eyes 14 days post-injection. Scale bars = 50 μm. (**b**) Quantification of retinal vasculature as vessel area per unit area of retina analyzed shows no difference between SH-11037 and vehicle control treatments. (**c**) Representative mean ERG responses. (**d**) Quantification of scotopic a- and b- waves and photopic b-wave shows no difference in retinal function (stimulus: scotopic = 2.5, photopic = 25 cd∙s/m^2^). *P* > 0.05, Student’s t-test. Mean ± s.e.m., n = 6 mice/treatment.

Given the effects of SH-11037 on angiogenesis *ex vivo* and in zebrafish development, we examined whether SH-11037 would cause regression of pre-existing retinal vasculature or damage to retinal function. Whole retina flatmounts were prepared 14 days after 100 μM SH-11037 or vehicle intravitreal injections and stained with isolectin B4 (Fig. 4a). No changes in the pre-existing retinal vessels were observed after SH-11037 treatment compared to the vehicle control (Fig. 4b). Moreover, electroretinography (ERG) was used to evaluate changes in the function of neural retina 14 days after 100 μM SH-11037 injections. Scotopic a- and b-waves, and photopic b-waves were not significantly different in SH-11037 treated eyes relative to the control eyes (Fig. 4c,d). These results demonstrate that SH-11037 does not interfere with the function of neural retina or the maintenance of normal retinal vasculature.

### SH-11037 significantly suppresses CNV lesion volume

**Figure 5:**
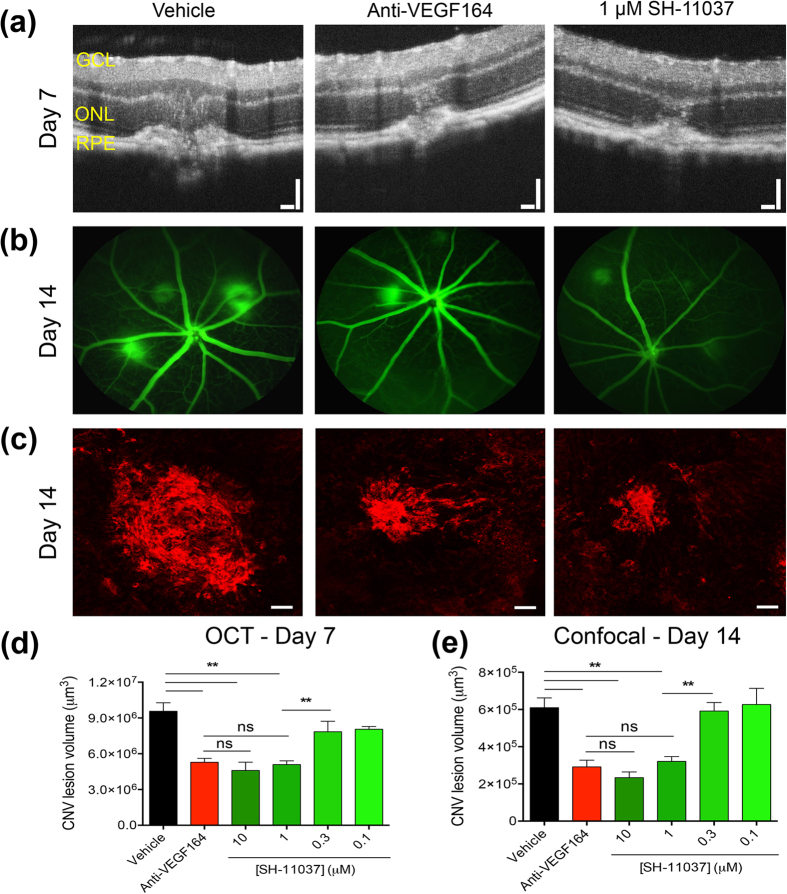
SH-11037 dose-dependently suppresses CNV lesion volume. (**a**) Representative OCT images obtained 7 days post-laser, showing CNV lesions of vehicle (left), anti-VEGF164 (middle), and 1 μM SH-11037 (right). Scale bars = 100 μm. (**b**) Representative images from fluorescein angiography 14 days post-laser. (**c**) Representative images from confocal microscopy for agglutinin-stained CNV lesions 14 days post-laser, scale bars = 50 μm. (**d**) Quantification of CNV lesion volumes from OCT images at day 7 using ellipsoid volume measurement^24^. (**e**) Quantification of CNV lesion volumes from Z-stack images at day 14 using ImageJ software. **P < 0.01, one-way ANOVA, Tukey’s post hoc tests, ns; non significant. Mean ± s.e.m., n = 12 eyes/treatment.

After confirming that SH-11037 was non-toxic, to examine the ability of this compound to reduce choroidal neovascularisation *in vivo*, we used the standard mouse L-CNV model^23^. Intravitreal injections of different SH-11037 concentrations (0.1, 0.3, 1, 10 μM) were given to C57BL/6J mice immediately after laser application (Fig. 5). The CNV lesion volumes in the SH-11037 treated eyes were significantly lower than those in vehicle treated eyes in a dose-dependent manner at 7 days post-laser, as monitored *in vivo* by optical coherence tomography (OCT) and measured by ellipsoid volume quantification^24^ (Fig. 5a,d). These decreases were comparable to those induced by an anti-VEGF164 antibody, which is a murine-optimized equivalent of bevacizumab, the standard of care in humans^25^. Additionally, fluorescein angiography revealed reduced leakiness of CNV lesions from SH-11037 and anti-VEGF164 treated eyes relative to the vehicle treatment (Fig. 5b). Confocal images of agglutinin-stained choroidal flatmounts revealed a reduction in CNV lesion size at 1 and 10 μM SH-11037 and anti-VEGF164 treated eyes compared to vehicle controls (Fig. 5c). Although there was no reduction in the CNV lesion volume compared to the vehicle control in eyes treated with SH-11037 at 0.1 and 0.3 μM, there was a dose-dependent reduction of CNV lesion volume of about 42% at 1 μM and 55% at 10 μM SH-11037 compared to the control eyes (*P* < 0.01) (Fig. 5e). Interestingly, these antiangiogenic effects were comparable to the anti-VEGF164 treatment that demonstrated about 50% inhibition of CNV lesion volume.

### SH-11037 cooperates with anti-VEGF therapy *in vitro* and *in vivo*

**Figure 6:**
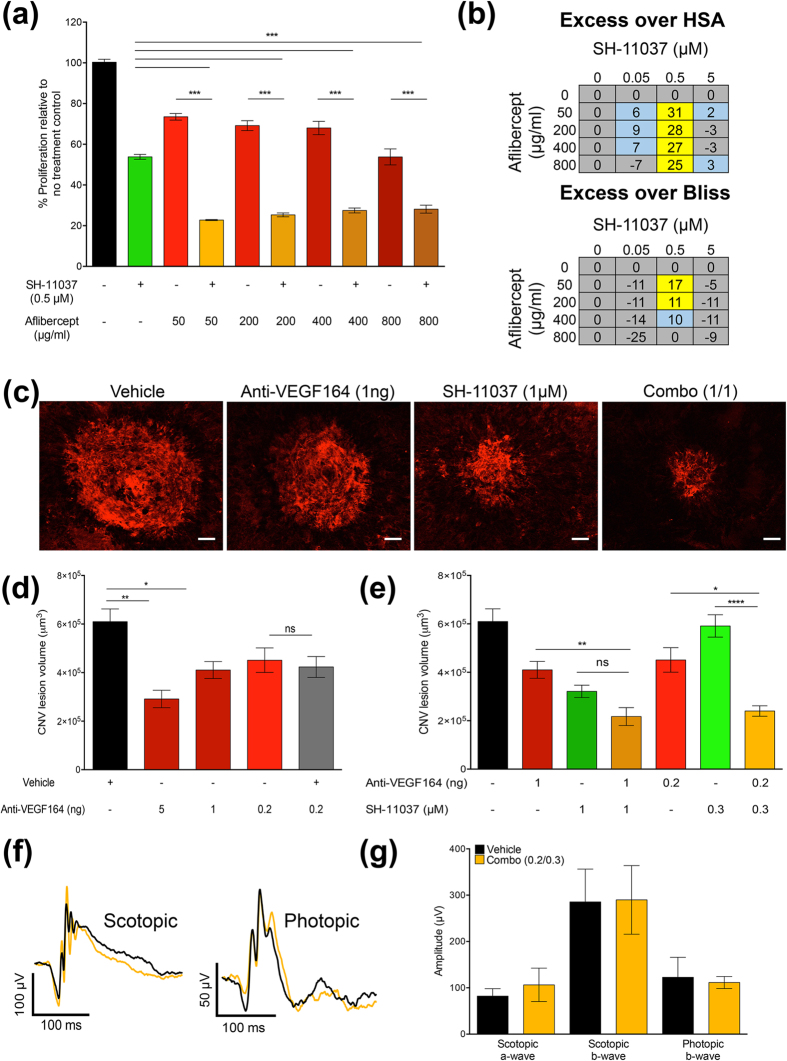
SH-11037 synergises with anti-VEGF therapy *in vitro* and *in vivo*. (**a**) The effects of 0.5 μM SH-11037, different concentrations of aflibercept, and SH-11037/aflibercept combinations on HREC proliferation were tested using an alamarBlue fluorescence assay, ***P < 0.001, one-way ANOVA with Tukey’s post hoc tests. Mean ± s.e.m., n = 3. Representative data from duplicate experiments. (**b**) Calculations of the excess % inhibition over highest single agent (HSA) activity and excess over Bliss additivity (see Methods). Values ≤ 0 are coloured in grey, values from 1–10 are in blue, values >10, indicating synergy, are yellow squares. (**c**) Representative images from confocal microscopy of agglutinin stained CNV lesions for combination treatment; 1 ng anti-VEGF/1 μM SH-11037 (Combo (1/1)) compared to individual SH-11037 and anti-VEGF164 treatments and vehicle control, scale bars = 50 μm. (**d**) Dose-dependent inhibition of the volume of CNV lesions by anti-VEGF164 injections. No difference was observed between anti-VEGF164 + vehicle compared to anti-VEGF164 alone (**e**) Quantification of CNV lesion volume shows a substitution of anti-VEGF164 by SH-11037. The combination of 1 ng anti-VEGF164 with 1 μM SH-11037 produces a similar effect to that observed by the 5 ng dose of anti-VEGF164 alone. The combination of 0.2 ng anti-VEGF164 with 0.3 μM SH-11037 (Combo (0.2/0.3)) is significantly different from individual treatments alone, and exceeds Bliss additivity and HSA by 36 and 37%, respectively, indicating synergy. Graphs in (**d**,**e**) are quantification of CNV lesion volumes from Z-stack images. *P <0.05, **P <0.01, ****P <0.0001, ns; non-significant, one-way ANOVA, Tukey’s post hoc tests. Mean ± s.e.m., n = 12 eyes/treatment. (**f**) Representative mean ERG responses of combo (0.2/0.3) and vehicle treatments. (**g**) Quantification of scotopic a- and b- waves and photopic b-wave shows no difference in retinal function (stimulus: scotopic = 2.5, photopic = 25 cd∙s/m^2^). *P* > 0.05, Student’s t-test. Mean ± s.e.m., n = 6 eyes/treatment.

Since many of the complications of anti-VEGF injections are concentration or dosing frequency dependent^26^,^27^, the possibility of reducing anti-VEGF dosage by combining with small molecules is highly desirable. We first tested whether SH-11037 and aflibercept (also known as VEGF Trap) would have a combined effect on the proliferation of HRECs using an alamarBlue fluorescence assay. Different concentrations of aflibercept (50, 200, 400, and 800 μg/ml) were tested alone and in combination with 0.5 μM SH-11037. Interestingly, HREC proliferation was significantly inhibited in the presence of combined treatments more than each treatment alone (Fig. 6a). To investigate the nature of the combined effect produced, excess over highest single agent (HSA) and excess over Bliss additivity were calculated (Fig. 6b). Values greater than zero observed in both analyses indicate synergistic effects of the tested aflibercept concentrations with 0.5 μM SH-11037.

Then, we tested the potential of SH-11037 and anti-VEGF164 combinations *in vivo* in the L-CNV mouse model. We first established a dose-response effect of intravitreal injections of SH-11037 and anti-VEGF164 separately (Figs 5e and 6d). Based on these results, a combination of the lowest fully active dose of SH-11037, 1 μM, and the suboptimal dose of anti-VEGF164, 1 ng/eye (Fig. 6c) was chosen. Analysis of confocal Z-stack images of agglutinin stained choroidal flatmounts revealed a significant reduction in neovascularisation with the combination therapy compared to anti-VEGF164 alone, *P* < 0.01 (Fig. 6e). Moreover, a combination of individually inactive doses of SH-11037 (0.3 μM) and anti-VEGF164 (0.2 ng/eye) caused a significant suppression of CNV lesion volume compared to SH-11037 and anti-VEGF164 alone, *P* < 0.0001, and *P* < 0.05, respectively (Fig. 6e), without interfering with retinal function as examined by ERG (Fig. 6f,g). Interestingly, this combination was synergistic, with percent inhibition of 37 and 36 in excess over HSA and Bliss additivity, respectively.

## Discussion

Angiogenesis is a highly regulated process that involves the formation of new blood vessels from existing ones, which is promoted by positive (proangiogenic) mediators such as VEGF^28^. Uncontrolled angiogenesis has been implicated in various pathological conditions, such as cancer and inflammation, and notably in blindness-causing diseases. Due to the existing limitations of anti-VEGF biologics^29^,^30^, and the fact that the pathological formation of blood vessels in the eye involves other angiogenic and inflammatory pathways^31^, the development of new small molecule inhibitors of ocular angiogenesis is crucial to complement the existing biologic therapies. Currently, there are no FDA approved small molecules for ocular neovascularisation. However, several promising small molecules are currently in Phase 1 or 2 clinical trials for the treatment of wet AMD such as squalamine, X-82 and PAN-90806 (www.clinicaltrials.gov)^32^. Interestingly, squalamine, which has been recently approved for a Phase 3 trial, is a natural aminosterol derived from dogfish shark cartilage^32^, and underwent several evaluation steps *in vitro* on endothelial cells^33^, *ex vivo* in an angiogenesis assay^33^, and *in vivo* in the oxygen-induced retinopathy (OIR) and L-CNV murine models^34^,^35^. Small molecules such as squalamine, and our compound, SH-11037, have the advantage of possible optimisation for topical administration over high molecular weight biologics that require localised drug delivery through intravitreal injection.

In the present study, we report the pharmacological activity and safety of SH-11037 in the context of ocular angiogenesis. Endothelial cells from different vascular beds have different physiological properties^36^. Therefore, we first sought to specifically evaluate the effects of SH-11037 in AMD-relevant microvascular tissues of the choroid *ex vivo*, taking advantage of the capability of the choroidal sprouting assay to measure microvascular angiogenesis in the choroid^37^. SH-11037 demonstrated a potent inhibitory concentration, which was consistent with the growth inhibitory concentration observed *in vitro* in retinal microvascular endothelial cells^18^.

In an initial experiment, SH-11037 suppressed retinal neovascularisation in the OIR model of ROP^18^. Interestingly, SH-11037 also demonstrated inhibition of ocular neovascularisation in the zebrafish. While 10 μM SH-11037 present in the embryo medium caused some oedema and cardiac toxicity on zebrafish larvae, these effects are commonly observed with testing antiangiogenic compounds in this model^22^, and it did not cause loss of pre-existing intersegmental vessels. Up to 100 μM SH-11037, which is at least 10 fold higher than the doses used for *in vivo* efficacy assessment, was not associated with short or longer-term signs of ocular toxicity in mouse retina, when the compound was injected intravitreally. It did not interfere with retinal function or the existing retinal vasculature, suggesting that SH-11037 specifically targets proliferating endothelial cells as previously shown^18^. However, although we did not observe any gross systemic effects of intravitreal SH-11037, the systemic toxicology of this compound remains to be evaluated.

In a subpopulation of human patients, anti-VEGF therapies can cause adverse effects in multiple organs including the eye. Some of these complications, such as retinal detachment and loss of neural retinal cells, are related to the doses of anti-VEGF treatment given to the patient^38^; thus, lowering anti-VEGF doses while maintaining the therapeutic efficacy would be beneficial^11^. Aflibercept, a fusion protein that consists of VEGF receptor-binding sequences fused to a segment of a human antibody backbone, is used in the clinic for the treatment of wet AMD^39^. Interestingly, SH-11037 produced combined effects with different concentrations of aflibercept in inhibiting the proliferation of HRECs, which were more pronounced than each treatment alone. Moreover, our *in vivo* data in the L-CNV model indicate that SH-11037 not only demonstrated a strong therapeutic potential for the amelioration of CNV as a single treatment, but also combined with the standard anti-VEGF164 antibody. Intriguingly, combining two individually inactive doses of SH-11037 and anti-VEGF164 produced a significant inhibition of L-CNV lesions that was comparable to the fully active dose of either treatment alone without affecting retinal function. SH-11037 and anti-VEGF combinations tested *in vitro* and *in vivo* appeared synergistic according to excess over HSA and Bliss additivity, two established methods of assessing synergy^40^, suggesting a VEGF-independent mode of action of SH-11037. However, the exact mechanism by which SH-11037 mediates its antiangiogenic effects is not yet known.

We have evaluated SH-11037’s pharmacological activity as an antiangiogenic small molecule in different models, from an *ex vivo* system of the choroid to a simple ocular angiogenesis assay in a whole organism in the zebrafish model, and finishing with a disease-relevant mouse model of CNV. Additionally, we extensively investigated the ocular toxicity of SH-11037 by retinal histology, markers of retinal damage, retinal vasculature and retinal function. SH-11037 might not be a suitable candidate for oral dosing due its limited water solubility. However, as a small molecule, it could potentially be optimised for topical applications, which would be appealing as a non-invasive local delivery route, while still minimising systemic side effects. Topical delivery could also enable frequent dosing. Alternatively, delayed-release formulations of this small molecule therapy for intravitreal injection might be possible if necessary, although our finding that a single injection of compound has an effect on L-CNV assessed two weeks later suggests a reasonable half-life. Determination of the pharmacokinetics of SH-11037 will be an essential step to further improve SH-11037 efficacy and route of administration.

Despite the power of anti-VEGF therapies, improvement of drug treatments for wet AMD and other neovascular eye diseases – and neovascularisation elsewhere in the body – will become necessary with increasing population age. With such promising therapeutic potential of this novel small molecule, extensive work is currently ongoing to unravel SH-11037’s mode of action and pharmacokinetic properties *in vitro* and *in vivo*.

## Methods

### Mice

All mouse experiments were performed in accordance with the guidelines of the Association for Research in Vision and Ophthalmology Statement for the Use of Animals in Ophthalmic and Visual Research and were approved by the Indiana University School of Medicine Institutional Animal Care and Use Committee. Wild-type female C57BL/6J mice, 6–8 weeks of age, were purchased from the Jackson Laboratory (Bar Harbor, ME, USA). Intraperitoneal injections of 17.5 mg/kg ketamine hydrochloride and 2.5 mg/kg xylazine mixture were used for anesthesia.

### Choroidal sprouting assay

Sprouting of choroidal layers was tested as previously described^37^. Briefly, 6–8 week old C57BL/6J mice eyes were enucleated immediately after euthanasia. Peripheral parts of the choroid/sclera layer were separated and cut into pieces. Choroid fragments were then placed on growth factor-reduced Matrigel^TM^ (30 μL/well; BD Biosciences, San Jose, CA, USA) in 24 well plates. The plates were then incubated at 37 °C for about 10 minutes to allow the Matrigel to solidify. Endothelial Growth Medium (EGM-2) was prepared by mixing the contents of an EGM-2 “Bullet Kit” (Cat no. CC-4176) with Endothelial Basal Medium (EBM) (Lonza, Walkersville, MD, USA). Penicillin-streptomycin and 2.5 mg/mL Plasmocin were added to the final medium and used for this assay. Medium (500 μL) was added to each well of the plate and incubated at 37 °C with 5% CO_2_ for 72 hours before any treatments were added. Medium was changed after 48 hours. SH-11037 synthesised as previously described^18^ and dissolved in DMSO was tested at 0.03, 0.3, and 1 μM concentrations for 48 hours. The final concentration of DMSO in each well was 0.2%. Trypan blue 0.4% solution (Fisher Scientific, Pittsburgh, PA, USA) was used to assess cell viability. Images were taken using an EVOS-fl digital microscope (AMG, Mill Creek, WA, USA) and data were analysed as the sprouting distance in four different directions using ImageJ software v. 1.48v (http://imagej.nih.gov/ij/).

### Zebrafish drug treatment and vessel quantification

*Tg*(*fli1:EGFP*) adult zebrafish (*Danio rerio*) were maintained as per standard husbandry practices with 14 h light/10 h dark cycle at 28 °C. Experiments were conducted in accordance with an ethical exemption approval granted by the University College Dublin animal research ethics committee. *Tg*(*fli1:EGFP*) zebrafish embryos were obtained through natural spawning and larvae staged based on morphological criteria. Larvae at two days post fertilisation (dpf) were placed in a 48 well plate with 5 larvae per well. Larvae were treated with 1, 2.5, 5, 7.5, and 10 μM SH-11037 in embryo medium with a final well volume of 400 μL. Larvae remained in treatment from 2–5 dpf at 28 °C. At 5 dpf, larvae were fixed overnight in 4% paraformaldehyde (PFA) at 4 °C, and subsequently washed in 1 × phosphate buffered saline (PBS). Fixed larvae were screened for overall morphological defects and a single lens from each larva dissected and the number of GFP positive primary HV and vessel branches were quantified using an Olympus SZX16 fluorescent microscope. The numbers of pre-developed intersegmental vessels from the trunk of the larvae were quantified to assess the effects of different treatment conditions on non-ocular vessels.

### Intravitreal injections

Injections were given to mice under anesthesia in a 0.5 μL volume using a 33-gauge needle. The needle was kept in place for 1 min to prevent the reflux of solution when the needle was removed. SH-11037 was dissolved in DMSO then diluted in PBS to a final concentration of 0.5% DMSO. Vehicle alone was used as control. Eyes were numbed with tetracaine solution before the injection, and triple antibiotic ointment was used immediately after the injection to prevent infection. A masked researcher undertook imaging and analysis to avoid bias.

### *In vivo* toxicity

Toxicity of intravitreally injected SH-11037 was initially assessed by histology and immunofluorescence. The presence of acute ocular toxicity was investigated by injecting SH-11037 at different concentrations (0.1, 1, 10, and 100 μM), while longer-term effects were tested by injecting 10 μM SH-11037. Mice were sacrificed 3 and 14 days post injections, respectively. Eyes were enucleated and fixed in 4% PFA overnight, and then the eyes were paraffin embedded and sectioned at 5 μm thickness by the Indiana University School of Medicine Histology Core. Mayer’s H&E staining was performed^41^, and retinal morphology quantified by calculating the ratio of A (the distance from the ganglion cell layer to the outer edge of the inner nuclear layer) to B (the distance form the ganglion cell layer to the outer edge of the outer nuclear layer) as previously described^42^.

For immunofluorescence, sections were deparaffinised with xylenes and ethanol series and boiled in citrate buffer for 5 minutes. Sections were blocked for one hour with 10% DAKO blocking buffer (DAKO, Carpinteria, CA, USA) in Tris-buffered saline (TBS), then probed overnight at 4 °C with primary antibodies diluted 1:400 in 10% DAKO diluent in TBS: GFAP (D1F4Q) and cleaved caspase 3 (D175) (Cell Signaling Technologies, Danvers, MA, USA) and MCP1 (Novus Biologicals, Littleton, CO, USA). Sections were then incubated with Alexafluor 555-conjugated goat anti-rabbit antibody (Life Technologies, Waltham, MA, USA) for 1 hour, 1:400 in TBS. TUNEL staining was performed using Click-iT TUNEL assay kit (Fisher Scientific, Pittsburgh, PA, USA) as per the manufacturer’s instructions after the citrate-washing step. Sections were washed with TBS, dehydrated through an ethanol series, and then mounted with Vectashield mounting medium with DAPI (Vector Labs, Burlingame, CA, USA). Images were taken with an LSM700 confocal microscope (Zeiss, Thornwood, NY, USA) with a 40× objective.

### Retinal electrophysiology and vasculature staining

ERGs were obtained from animals 14 days post intravitreal injection of 100 μM SH-11037, 0.2 ng mouse anti-VEGF164 neutralizing antibody (R&D Systems, Minneapolis, MN, USA) + 0.3 μM SH-11037, or vehicle control and performed as previously described^43^. Briefly, scotopic rod recordings were performed on overnight dark-adapted mice, with 10 increasing light intensities of white light, and responses were recorded with a visual electrodiagnostic system (UTAS-E 2000; LKC Technologies, Gaithersburg, MD, USA). Stimuli were presented at intensities of 0.025, 0.25, and 2.5 log cd∙s/m^2^ at 10-, 20-, and 30-second intervals, respectively. Ten responses were recorded and averaged at each light intensity. Photopic cone recordings were undertaken after mice were light adapted to a white background light of 100 cd∙s/m^2^ for 8 min. Recordings were performed with four increasing flash intensities from 0, 5, 10 and 25 log cd∙s/m^2^ in the presence of a constant 100 mcd∙s/m^2^ rod suppressing background light. The b-wave amplitude was determined from a-wave trough to b-wave peak, behind the last prominent oscillatory potential. Preparation of retina flatmounts and staining of pre-existing vasculature was performed as previously described^18^. The retinal vessel area in the superficial plexus was measured from four random fields from four different sections per eye using Adobe Photoshop software and data are presented as the retinal vessel area per unit area of retina analyzed.

### Laser-induced choroidal neovascularisation

Laser photocoagulation was performed as previously described^24^,^44^. Briefly, eyes were dilated using 1% tropicamide, then underwent laser treatment using 50 μm spot size, 50 ms duration, and 250 mW pulses of an ophthalmic argon green laser, wavelength 532 nm, coupled to a slit lamp. A coverslip was used to allow viewing of the posterior pole of the eye. Each eye received 3 laser burns centred around the optic nerve at 12, 3, and 9 o’clock positions. The appearance of a bubble at the site of laser application was used to identify laser-induced damage of Bruch’s membrane. Lesions in which bubbles were not observed were excluded from the study. The effect of SH-11037 on the L-CNV model was tested using final estimated vitreal concentrations of 0.1, 0.3, 1, or 10 μM SH-11037. Anti-mouse VEGF164 was tested at 0.2, 1, and 5 ng/eye. Vehicle (PBS + 0.5% DMSO) was used as a negative control. All injections were given a single time, immediately after laser treatment.

### Optical coherence tomography and fluorescein angiography

OCT was performed at the indicated times using the Micron III intraocular imaging system (Phoenix Research Labs, Pleasanton, CA, USA). Before the procedure, eyes were dilated with 1% tropicamide solution (Alcon, Fort Worth, TX, USA) and lubricated with hypromellose ophthalmic demulcent solution (Gonak) (Akorn, Lake Forest, IL, USA). Mice were then placed on a custom heated stage that moves freely to position the mouse eye for imaging. Several horizontal and vertical images were taken per lesion to allow calculation of CNV lesion volume. Three-dimensional quantification of CNV lesion volumes was performed using an ellipsoid quantification method as previously described^24^. Fluorescein angiography was performed 14 days post laser by intraperitoneal injection of 50 μL of 25% fluorescein sodium (Fisher Scientific, Pittsburgh, PA, USA). Fundus images were taken using the Micron III system and Streampix software.

### Choroidal flatmount and analysis

Choroidal flatmount preparation, staining, and imaging were undertaken as previously described^24^. ImageJ software was used to analyze *Z*-stack images; the summation of the whole stained area in each section, multiplied by the distance between sections (3 μm) was used as an index for the CNV lesion volume. The volumes of the 3 lesions in each eye were averaged and considered as an n = 1 for statistical analysis.

### *In vitro* proliferation assay and synergy analysis

The proliferation of HRECs was monitored by an alamarBlue fluorescence assay as described previously^45^. Briefly, 2,500 cells in 100 μL Endothelial Basal Medium (EBM) (Lonza) in the presence of 50 ng/mL recombinant human VEGF165 (Biolegend, San Diego, CA, USA), were incubated in 96-well clear bottom black plates for 24 hours followed by 48 hours incubation with either 0.5 μM SH-11037, different concentration of aflibercept (Eylea, Regeneron) (50, 200, 400, 800 μg/mL), or combination treatment. At the end of the incubation, 11.1 μL of alamarBlue reagent was added and 4 hours after, fluorescent readings were taken on a Synergy H1 plate reader (Biotek, Winooski, VT, USA) with excitation and emission wavelengths of 560 nm and 590 nm respectively. Data were analyzed using GraphPad Prism software (v. 6.0).

Synergy analysis was performed as previously described^40^. The excess inhibition over the HSA represents the inhibition of the combination mixture over the highest effect seen with either single agent alone at the same concentration as in the mixture. Bliss additivity calculates the predicted combined response *C* for two single compounds with effects *A* and *B* as: *C*  =  *A* + *B* − *A* * *B*. The predicted effect *C* was then subtracted from the experimentally observed percent inhibition to generate excess over Bliss values, where positive values indicate synergistic effects.

### Statistical analyses

Statistical analyses were performed with GraphPad Prism 6 software. Student’s *t*-tests were used to compare retinal thickness measurements and ERG parameters. Repeated measures two-way ANOVA with Dunnett’s *post hoc* test was used for choroidal sprouting distance measurements. One-way ANOVA with Dunnett’s *post hoc* test was used for analysis of zebrafish data. For all other experiments, one-way ANOVA was used with Tukey’s *post hoc* tests to compare between treatments. *P* values < 0.05 were considered statistically significant.

## Additional Information

**How to cite this article**: Sulaiman, R. S. *et al.* A novel small molecule ameliorates ocular neovascularisation and synergises with anti-VEGF therapy. *Sci. Rep.* **6**, 25509; doi: 10.1038/srep25509 (2016).
